# Expression changes of ionic channels in early phase of cultured rat atrial myocytes induced by rapid pacing

**DOI:** 10.1186/1749-8090-8-194

**Published:** 2013-09-29

**Authors:** Qiang Ji, Hua Liu, Yunqing Mei, Xisheng Wang, Jing Feng, Wenjun Ding

**Affiliations:** 1 Department of Thoracic Cardiovascular Surgery of Tongji Hospital of Tongji University Shanghai, 389 Xincun Rd, Shanghai 200065, P.R. China; 2 Department of Cardiovascular Surgery of Zhongshan Hospital of Fudan University Shanghai, 180 Fenglin Rd, Shanghai 200032, P.R. China

**Keywords:** Atrial fibrillation, Atrial myocyte, Rapid pacing, Ionic channel, Remodeling

## Abstract

**Background:**

Recent studies have demonstrated that atrial electrical remodeling was an important contributing factor for the occurrence, persistence and maintenance of atrial fibrillation. The expression changes of ionic channels, especially L-type calcium channel and potassium channel Kv4.3, were the important molecular mechanism of atrial electrical remodeling. This study aimed to observe the expression changes of ionic channels in a rapid paced cell model with primary cultured atrial myocytes.

**Methods:**

The primary rat atrial myocytes were cultured, characteristics of the cultured myocytes were observed with light microscope and the cell phenotype was harvested by immunocytochemical stain to detect α-actin. The cellular model of rapid pacing was established with primary cultured atrial myocytes. The expressions of L-type calcium channel α_1_c and potassium channel Kv4.3 in cultured atrial myocytes were detected by immunocytochemistry, reverse transcription polymerase chain reaction and Western blot after rapid pacing.

**Results:**

The primary rat atrial myocytes were isolated and cultured successfully, and used for following experiment by identification of activity and purity. Cellular model of rapid electrical field pacing was established successfully. There is no significant difference in cell activity after pacing compared to that before pacing by 3-[4, 5-dimethylthiazol-2-y1]-2, 5-diphenytetrazolium bromide assay, and cell degeneration can be observed by transmission electron microscope. The mRNA expression of L-type calcium channel α_1_c started to reduce after 6 h of rapid pacing and continued to decline as pacing continued. Protein expression changes were paralleled with decreased mRNA expression of the L-type calcium channel α_1_c. The mRNA expressions of potassium channel Kv4.3 were not altered within the first 6 h, but after 12 h, mRNA expressions were reduced. Longer pacing periods did not further decrease mRNA expression of potassium channel Kv4.3. Protein expression changes were paralleled with decreased mRNA expression of potassium channel Kv4.3.

**Conclusions:**

Rapid paced cultured atrial myocyte model was established utilized primary cultured atrial myocytes and this model can be used for studying the early electrical remodeling in atrial fibrillation. Expressions of L-type calcium channel α_1_c and potassium channel Kv4.3 were both reduced at different levels in early phase of rapid pacing atrial myocytes. It implicates the occurrence of ionic channel remodeling of atrial myocytes.

## Background

Atrial fibrillation (AF) is the most common arrhythmias, increases with age, and presents with a wide spectrum of symptoms and severity [1]. Because of impaired atrial pump function and ventricular filling, AF may decrease the cardiac output. In addition, AF is an important risk factor for thrombosis and emboli. It is estimated that more than 15% of stroke is caused by AF [2]. The pathophysiologic mechanism underlying the occurrence, development and maintenance of AF is still poorly understood, which makes the treatment of AF a challenge for clinicians.

The main characteristics of AF are the changes in atrial electrophysiology and structure, which is called remodeling. Recent studies [3] reported that atrial remodeling, including electrophysiological and structural remodeling, played a key role in the development, maintenance and recurrence of AF. Electrical remodeling occurs earlier, and results in the shortening of wavelength of intra-atrial re-entry and increase in the number of re-entrant cycle, which in return causes AF maintenance. Some studies [4-7] have shown that the changes in ion currents, including K^+^ current, Ca^2+^ current, Na^+^ current, etc., are the important basis for early electrical remodeling in AF. Transient outflux K^+^ current (Ito) is the basis of early rapid repolarization, and the potassium channel Kv4.3 is the main contributor to Ito. Calcium channel current (ICa) is the main part of action potential and excitation function of myocardial cells. There are a variety of Ca^2+^ channel proteins in the myocardium, and the voltage-dependent calcium channels are divided into L- and T-type channels, in which influx Ca^2+^ current produced by L-type calcium channel plays an important role in the regulation of human atrial frequency-dependent action potential duration (APD) and endocardial return percentage (ERP) [8].

Some studies [7,9-11] have shown that the changes in the expression of ion channels, especially L-type calcium channel and potassium channel Kv4.3, are the molecular basis of electrical remodeling secondary to AF. However, treatment targeting above changes usually fails to improve the AF and its prognosis. Thus, the mechanisms underlying these changes are needed to be elucidated. The present study aimed to explore the method of culturing primary atrial myocardial cells *in vitro* as well as to establish an *in vitro* model of rapid electric pacing of cultured atrial myocardial cells. Then, the changes in the expressions of L-type calcium channel and potassium channel Kv4.3 were investigated in the early phase of rapid pacing.

## Methods

### Animals and materials

Wistar rats aged 2 weeks were purchased from the Experimental Animal Center of Shanghai Tongji Hospital. BB5060UV CO_2_ incubator (Germany), AlphaImager EP gel imaging system (NatureGene Corp., American), DU800 UV-visible spectrophotometer (Beckman Company, American), Leica DMI4000B inverted fluorescent microscope (Germany), PTC-100-96HV thermal cycler (American), CHK-213 Olympus® biological microscope (Japan), DYY-III24D dual vertical electrophoresis bath (Beijing Liuyi Company, China), BL-420E + biological functional experimental system (Chengdu Taimeng Science and Technology Ltd., China); collagenase type II and 5-bromodeoxyuridine (Brdu, Sigma, American), mouse anti-rat sarcomeric actin antibody (Wuhan Boster Bio-engineering Limited Company, China), fluorescein isothiocyanate-labeled (FITC-labeled) goat anti-mouse IgG (Beijing Zhongshan Company, China), rabbit anti-rat L-type calcium channel and potassium channel KV4.3 polyclonal antibodies (Chemicon company, American), goat anti-rabbit HRP- conjugated IgG (Beijing Zhongshan Company, China), Strept Avidin-Biotin Complex (SABC) immunohistochemical staining kit and diaminobenzidine (DAB) kit (Wuhan Boster Bio-engineering Limited Company, China), reverse transcription PCR (RT-PCR) kit (Progema, American), Coomassie Brilliant Blue R-250 and methylenebisacrylamide (Sigma, American), polyvinylidene difluoride (PVDF) membrane (Roche, Germany); PCR primer (Shanghai Boya biological Engineering Company, China) and PCR markers (Beijing Tianwei bio-engineering company, China) were used in the present study.

### Isolation and culture of myocardial cells

The isolation and culture of myocardial cells were conducted according to previously reported by Benardeau et al. [12] with modification (differential adherence method and drug intervention (Brdu) were adopted in this study to improve the purity of atrial myocardial cells). In brief, rats were fixed in a supine position. After sterilization, an incision was made along the right edge of the sternum and the chest wall was removed. The heart was collected rapidly, and then washed in D-hank’s solution to remove the blood. Then, the left and right atriums were collected and washed with a serum free medium. Under an aseptic condition, the right atrial appendage was trimmed into blocks with about 1 mm in diameter. These blocks were digested with 0.08% trypsin at 37°C for 5 min. The digestion was stopped by addition of serum-containing medium. This solution was allowed to keep at temperature for deposition, and the supernatant was tranferred into an aseptic container, filtered through a 100-mesh filter and then tranferred into a centrifuge tube. Digestion was done thrice. Finally, the cell suspension was treated with 0.1% collagenase at 37°C for 15 min. After pipetting for 1–2 min, the supernatant was collected, filtered through a 100-mesh filter and transferred into a centrifuge tube. The cell suspension was centrifuged at 1000 × g for 5 min at 4°C. The supernatant was removed, and the cells were washed with DMEM by centrifugation. Then, single cell suspension was prepared with DMEM containing 10% FBS. The cell density was adjusted to 1 × 10^8^/L and cells were then seeded into flasks followed by incubation at 37°C in an environment with 5% CO_2_ for 2 h. The cells was separated by means of their differential adherent properties to glass. The cell suspension was added to the plates containing coverslips, and cells were maintained in medium containing 0.1 mmol/L BrdU. The medium was refreshed 24 h later with BrdU free DMEM. The shape, time to adherence of cells, time to beating of these cells, and beating frequency were observed under an inverted microscope.

### Rapid stimulation of atrial myocardial cells and detection of action potential

When the cell confluence reached about 80%, the plates were placed in an electric field and stimulated with a BL-420E + biological functional experimental system (600 times/min, 1.5 V/cm) for 24 h. The survival rate of cells before and after the fast electric pacing was detected by 3-[4,5-dimethylthiazol-2-y1]-2,5-diphenytetrazolium bromide (MTT), the action potential duration at 50% repolarization was recorded with a patch clamp at the whole cell mode.

### Immunohistochemistry

Cells were harvested and fixed in 4% paraformaldehyde for 20 min followed by fluorescence immunohistochemistry. Cells were washed in 0.01 mmol/L PBS and then treated with mouse anti-rat α-actin at 4°C overnight. After washing thrice (5 min for each) with PBS, cells were treated with rat anti-mouse IgG-FITC at 37°C for 1 h). Cells were then observed under a fluorescence microscope, and representative photographs were captured.

### Preparation of samples for transmission electron microscopy and observation

Before and after stimulation, monolayer cells were collected and transferred into EP tube and re-suspended in pre-cold PBS followed by centrifugation at 1000 r/min for 5 min. The supernatant was removed and cells were fixed in 2% glutaraldehyde for 2 h and then in 1% tetroxide osmium for 2 h. After dehydration in a series acetone solution, cells were embedded in epoxy resin 618. Ultrathin sections were prepared and stained with uranium acetrate and lead citrate. Observation was done by transmission electron microscopy.

### Detection of L-type calcium channel α1c and potassium channel Kv4.3 expression by immunocytochemistry

Cells were washed in PBS, fixed in 4% paraformaldehyde for 30 min, washed in PBS thrice (2 min for each), treated with 3% hydrogen peroxide for 20 min at room temperature, washed in PBS thrice (2 min for each), blocked in 5% BSA at room temperature for 30 min and then treated with rabbit anti-rat L-type calcium channels α1c polyclonal antibody or potassium channel Kv4.3 polyclonal antibody (1:200). In the negative control, the primary antibody was replaced with PBS (4°C incubation overnight). After washing in PBS thrice (2 min for each), cells were treated with goat anti-rabbit HRP-conjugated IgG (1:1000) at room temperature for 1 h, washed in PBS thrice (2 min for each), incubated with SABC at room temperature for 30 min and washed in PBS thrice (5 min for each). Visualization was done with DAB followed by mounting and observation under microscopes.

### Detection of L-type calcium channel α_1_c and potassium channel Kv4.3 expression by RT-PCR and Western blot assay

The mRNA expressions of L-type calcium channel α1c and potassium channel Kv4.3 were detected by RT-PCR. The primers for L-type calcium channel α1c and potassium channel Kv4.3 were showed in Table 1. The protein expressions of L-type calcium channel α1c and potassium channel Kv4.3 were detected by western blot assay. Total protein was extracted followed by determination of protein concentration by using Bradford method. After SDS-PAGE, proteins were immunoblotted and visualization was done with DAB.

**Table 1:** **Primers for α**_**1**_**c, Kv4.3 and β-actin**

	**Length (bp)**	**Sequence**	**Annealing temperature**
α_1_c	324	5′-ATGGAGGCTGGAGCCCAGATTGA-3′	61.3°C
		5′-GACATTGAGGTCCGCACCGAAGG-3′	
Kv4.3	386	5′-GCAGCAACCTGAAATCTGAAACT-3′	56.1°C
		5′-GATAAGCAATGAACCCATCTCCA-3′	
β-actin	453	5′-TGAGAGGGAAATCGTGCGTGAC-3′	53.9°C
		5′-ATCTGCTGGAAGGTGGACAGTGAG-3′	

bp, base pair; α_1_c, L-type calcium channel α_1_c; Kv4.3, potassium channel Kv4.3.

## Results

### Culture and identification of atrial myocardial cells

**Figure 1:**
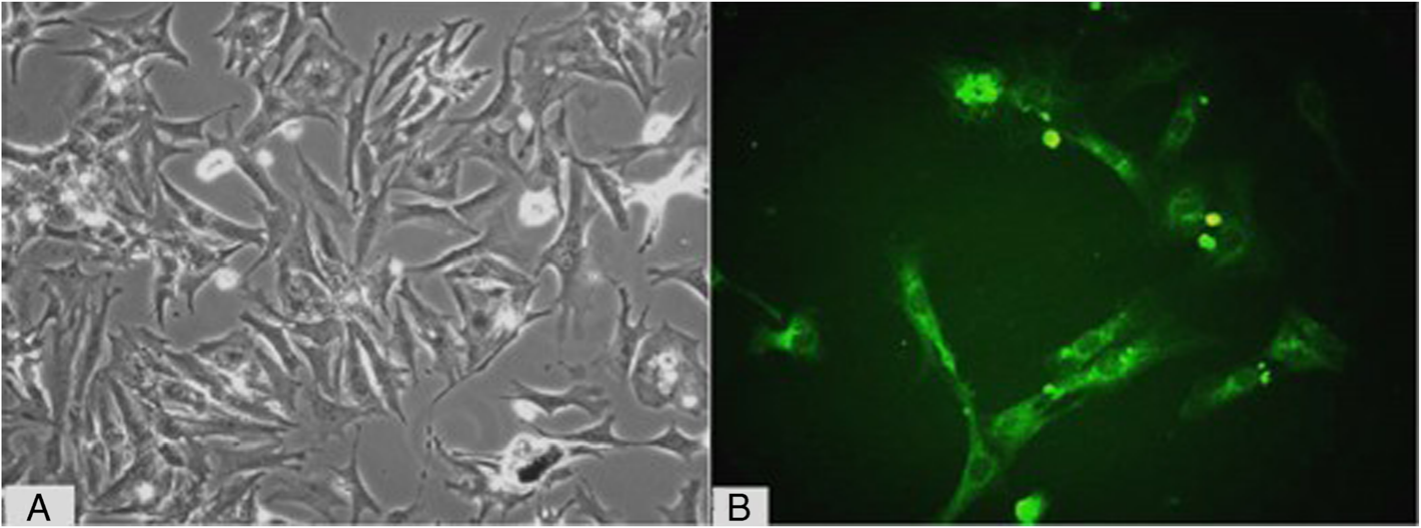
**Culture and identification of atrial myocardial cells (200×).** (**A**: Cultured atrial myocardial cells on day 4; **B**: Cultured cell presenting positive α-actin stain).

On day 4, the cultured atrial myocardial cells presented with various forms (Figure 1A). Under an inverted microscope, these cells were mainly rod, spindle-shaped, triangular or irregular. When the cell confluence reached 70%, the amount of these cells was about 2-3 × 10^9^/L. Most cells began to present with regular impulse at 24–48 h after culture. Immunocytochemistry showed more than 90% of cultured cells were positive for α-actin, which further confirmed that these cells were atrial myocardial cells (Figure 1B).

### Electrophysiological characteristics of cultured atrial myocardial cells

After stimulation by electrical field for 24 h, the APD50 of these cells (n = 10) was recorded with a patch clamp at the whole-cell mode. The APD50 was 64.2 ± 4.6 ms before stimulation and 56.6 ± 4.1 ms after stimulation, showing significant difference (p < 0.05).

### Ultrastructure of cells before and after stimulation

**Figure 2:**
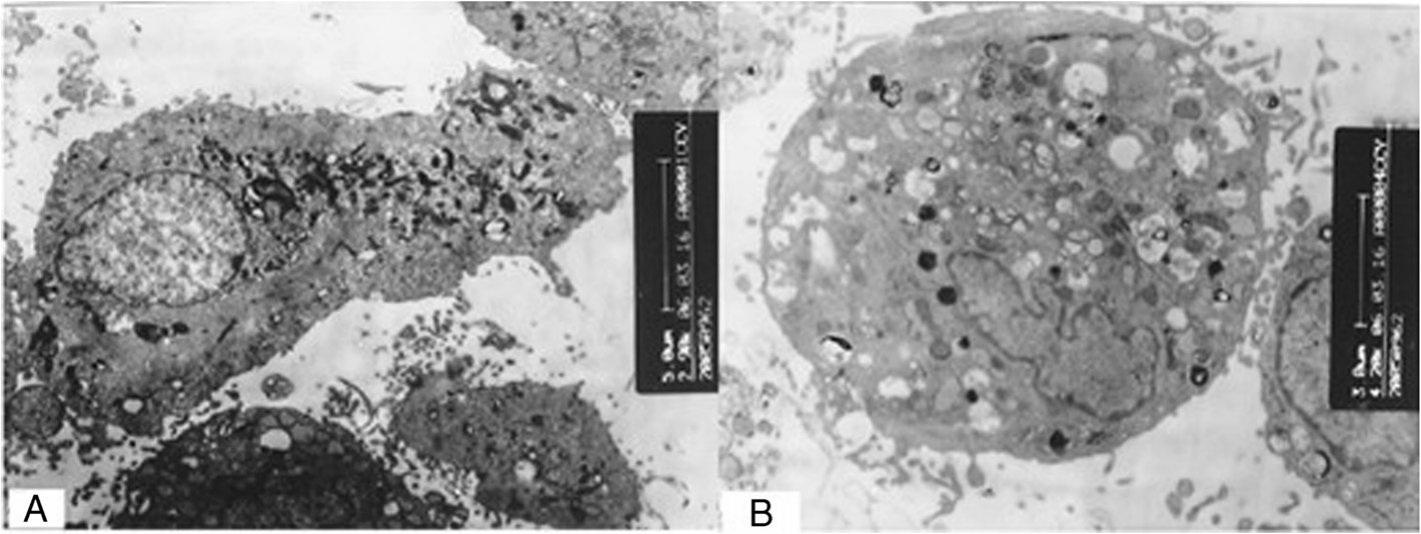
**Ultrastructure of cells before and after stimulation (14000×).** (**A**: Atrial myocardial cells before stimulation; **B**: Atrial myocardial cells after stimulation).

After stimulation, myolysis, vacuolization and karyopycnosis were observed in these atrial myocardial cells under a transmission electron microscope (Figure 2).

### Detection of potassium channels Kv4.3 and L-type calcium channel α_1_c by immunocytochemistry

**Figure 3:**
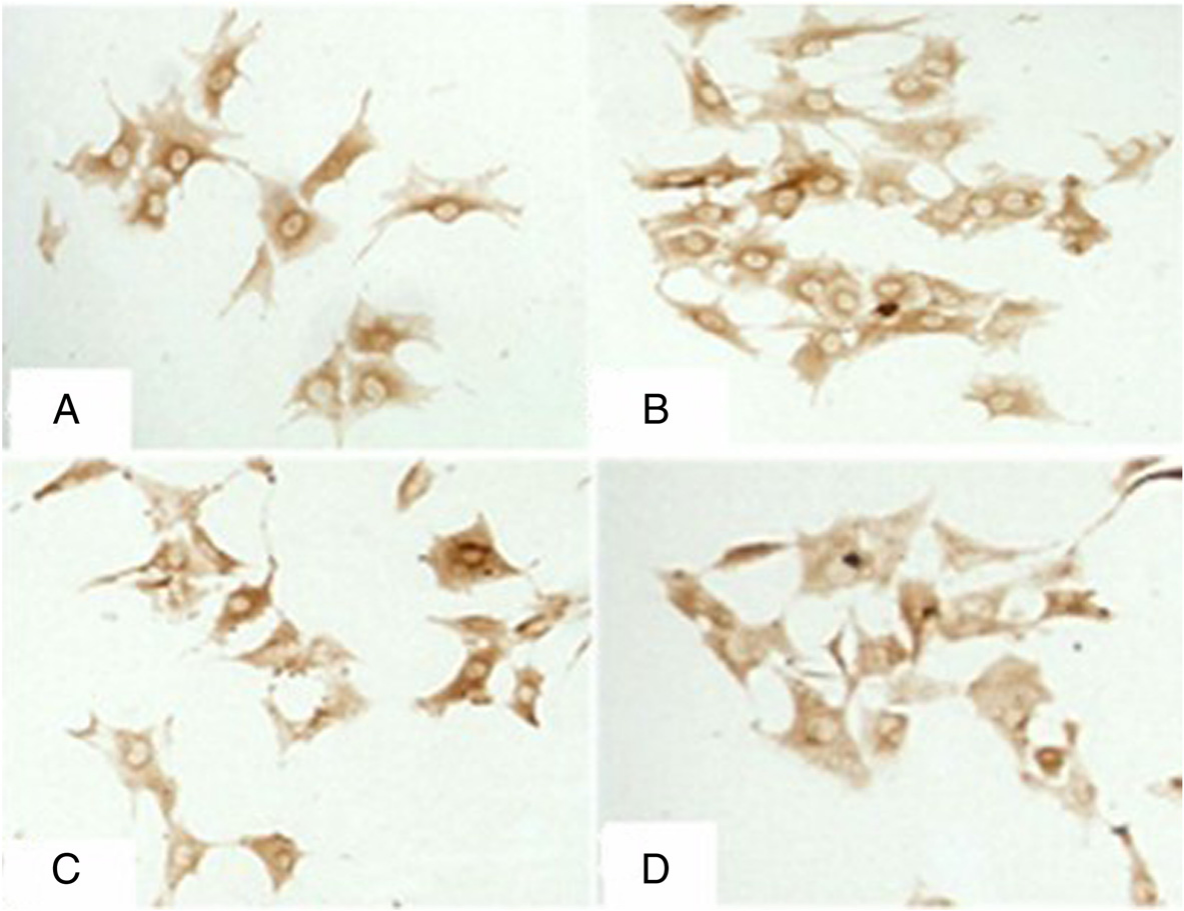
**Expression of Kv4.3 by immunocytochemistry (200×).** (**A**: Expression of Kv4.3 before pacing; **B**: Expression of Kv4.3 at 3 h after pacing; **C**: Expression of Kv4.3 at 12 h after pacing; **D**: Expression of Kv4.3 at 24 h after pacing).

**Figure 4:**
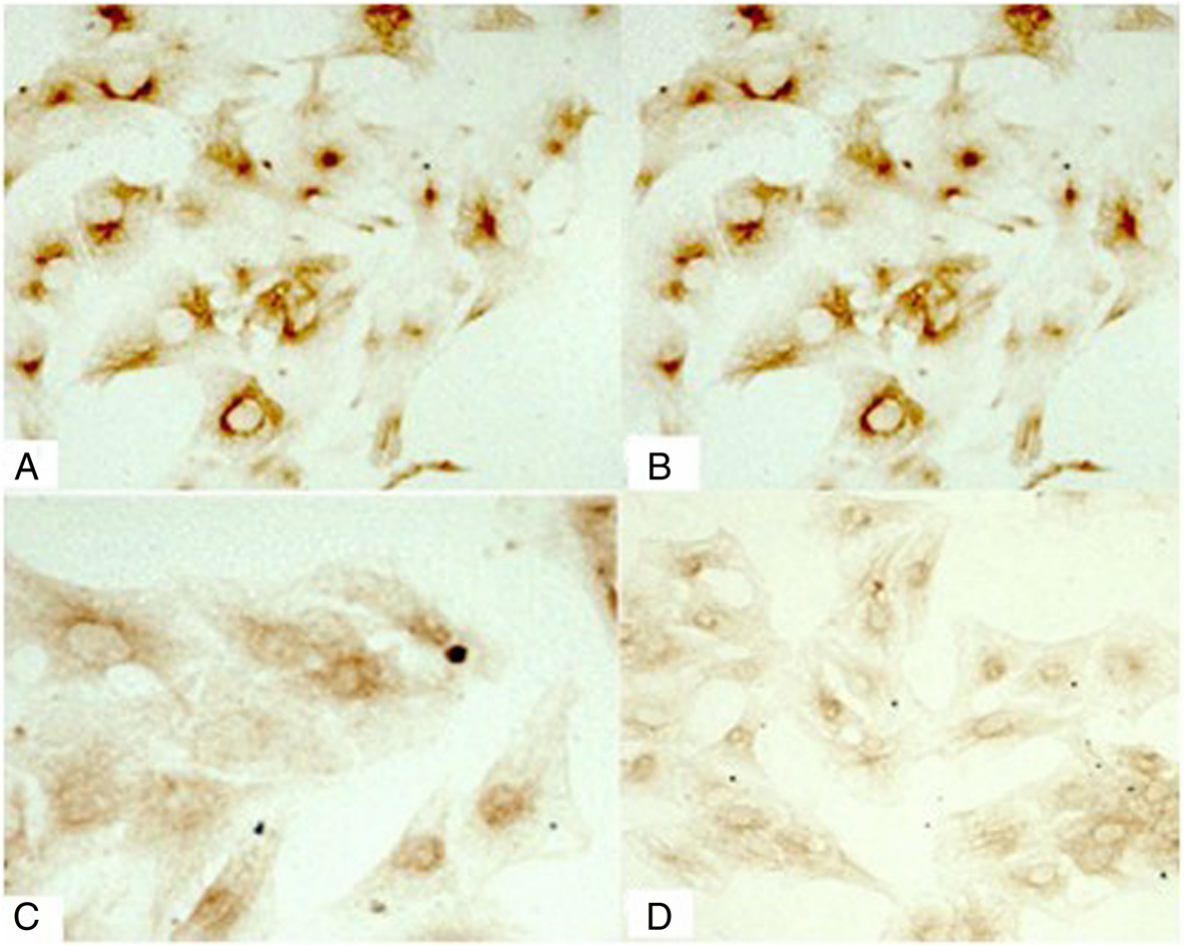
**Expression of L-type calcium channel α**_**1**_**c by immunocytochemistry (200×).** (**A**: Expression of L-type calcium channel α_1_c before pacing; **B**: Expression of L-type calcium channel α_1_c at 3 h after pacing; **C**: Expression of L-type calcium channel α_1_c at 12 h after pacing; **D**: Expression of L-type calcium channel α_1_c at 24 h after pacing).

The background was clear in all sections. There was no staining in the negative control group, and positive cells presented with brown granules in cells. At 3 h after pacing, the expression of L-type calcium channel α_1_c and potassium channel Kv4.3 remained unchanged when compared with that before pacing. The expression of potassium channel Kv4.3 at 12 h and 24 h was slightly lower than that before pacing, while the expression of L-type calcium channel α_1_c at 12 h and 24 h was significantly lower than that before pacing (Figures 3 and 4).

### Changes in mRNA expression of L-type calcium channel α_1_c

**Figure 5:**
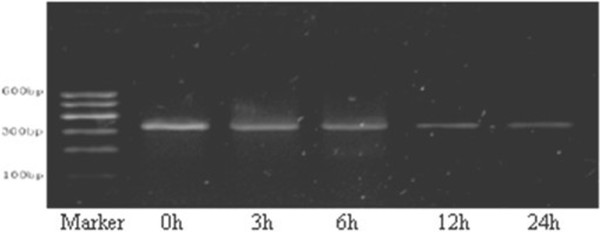
**mRNA expression of L-type calcium channel α**_**1**_**c.**

After rapid pacing for 6 h, the mRNA expression of L-type calcium channel α_1_c showed a reducing trend and further decreased over time, The mRNA expression of L-type calcium channel α1c at 6 h (1.14 ± 0.11), 12 h (1.03 ± 0.08) and 24 h (0.95 ± 0.10) was significantly reduced when compared with that before pacing (1.18 ± 0.13) (n = 5) (Figure 5).

### Changes in protein expression of L-type calcium channel α_1_c

**Figure 6:**
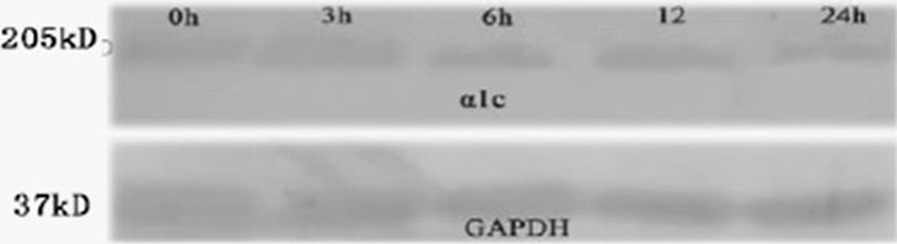
**Protein expression of L-type calcium channel α**_**1**_**c.**

The changes in protein expression of L-type calcium channel α_1_c were similar to those in its mRNA expression. When compared with before rapid pacing (1.01 ± 0.15), the protein expression of L-type calcium channel α_1_c was markedly reduced at 6 h (0.97 ± 0.17), 12 h (0.86 ± 0.13) and 24 h (0.80 ± 0.11) (n = 5) (Figure 6).

### Changes in mRNA expression of potassium channels Kv4.3

**Figure 7:**
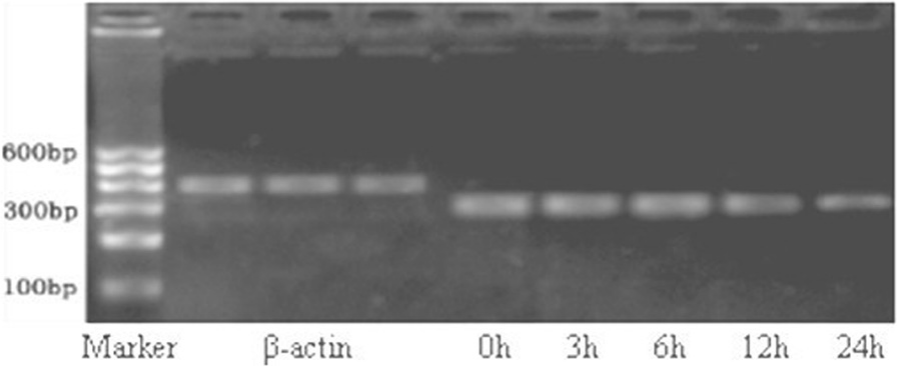
mRNA expression of Kv4.3.

The mRNA expression of potassium channels Kv4.3 began to decrease at 12 h after pacing, but thereafter remained stable and didn’t further reduce over time (Figure 7).

### Changes in protein expression of potassium channel Kv4.3

**Figure 8:**
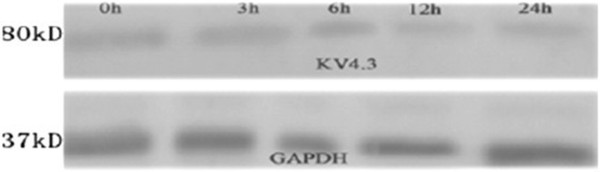
Protein expression of Kv4.3.

The relative protein expression of potassium channel Kv4.3 was: 0.56 ± 0.12, 0.56 ± 0.09, 0.55 ± 0.13, 0.46 ± 0.11 and 0.45 ± 0.08 at 0, 3, 6, 12 and 24 h after pacing, respectively (n = 5). The protein expression of potassium channel Kv4.3 at 12 h and 24 h was significantly lower than that at 0, 3 and 6 h (Figure 8).

## Discussion

Currently, most studies on AF are carried on patients with AF or in animal models. Although good results have been acquired, there are still some limitations, such as inability to precisely interfere, susceptibility to influence by many factors and ethical concerns [13]. As the maturation of culture of myocardial cells, primary myocardial cells can be used as an *in vitro* model for the study of pathophysiology of some cardiac diseases. The primary myocardial cells as an in vitro model has the following advantages: ① The enzyme induced damage (damage to proteins in cell membrane including receptors and ion channels) during the separation may restore completely after culture [13]. ② The cell culture can last for a long time. Studies showed that the vitality of myocardial cells undergoing acute separation can only be maintained up to 8–10 h, and that of cultured myocardial cells can be maintained for a few days, or even several weeks [14]. In addition, the *in vitro* study can avoid the influence of numerous factors in *in vivo* studies. ③ The molecular biological techniques can be used to detect the expression of proteins in myocardial cells alone. These cells maintain favorable vitality even the expression of these protein changes with the help of molecular biological technology [15,16]. ④ For Human and primate, the source of myocardial tissue from is limited. Thus, the cultured cells are a good choice. ⑤ The technique of storage of cells is mature. Thus, to establish an *in vitro* AF model with myocardial cells is very important.

The difficulty in sample collection, small number of collected cells And the technique for Cell separation are the major barriers in primary atrial myocardial cell culture. Mature atrial myocardial cells are regarded as terminally differentiated cells and are susceptible to damage by ischemia, hypoxia, enzymes, pH and mechanical stimulation which may ultimately affect the activity and amount of cells. In addition, the separation of these cells is difficult due to strong physical connection via the intercalated disk and extracellular matrix among cells. Moreover, the atrial myocardial cells are isolated under a calcium-free environment and thus intolerant to calcium. Under the physiological conditions, cells may present with contraction in the presence of calcium, which may result in change in the morphology of these cells. Thus, it is extremely difficult for separation and culture of atrial myocardial cells. In this study, the culture of primary atrial myocardial cells was done according to the method described by Benardeau et al. with modification [12]. In the separation and culture of atrial myocardial cells, we have following experience: ① The age and body weight should be taken into account in selection of rats. Rat aged about 2 weeks and weighing 30 g are preferred. Under this condition, the collection of atrial myocardial cells is relatively easy, the vitality of these cells is improved and the purification is more convenient. ② The trypsin may cause damage to these cells, and thus the trypsin concentration should be as low as possible. According to our experience, the trypsin concentration is usually 0.06-0.08%. The duration of digestion should not be too long. Digestion can be performed with short duration for sever times. ③ Brdu can significantly inhibit the growth of fibroblasts in the skeletal muscle and myocardium, but has no influence on or toxicity to myocardial cells. In addition, culture of myocardial cells with Brdu may increase the purity of myocardial cells from 45-50% to 85-90% [17]. Consequently, differential adherence method and drug intervention (Brdu) are crucial to improve the purity of atrial myocardial cells [18].

A rapid pacing model is established using primary myocardial cells (atrial myocardial cells or ventricular myocardial cells), which can also mimic the *in vivo* rapid pacing. In addition, electrophysiological detection, pharmacological intervention and molecular biological intervention are more convenient in primary myocardial cells [19]. In the present study, the separation, purification, culture and identification of atrial myocardial cells were conducted, and then these cells were used to establish a model of rapid pacing with electric field. After 24-h rapid pacing, the cell morphology remained unchanged, and the frequency of cell pacing was slightly faster. MTT chromatometry also demonstrated that the cell viability after rapid pacing was comparable to that before pacing. However, under a transmission electronic microscope, aggregation of glucogen, karyopycnosis, and vacuolar changes were observed. These changes were consistent to those in *in vivo* rapid pacing models and other *in vitro* rapid pacing models [5].

During the electrical remodeling after AF, the shortening of APD and ERP is secondary to the functional feedback of ion channel and becomes a characteristic in the early phase. However, in the late phase, the expression of L-type calcium channels and potassium channel Kv4.3 reduced resulting in changes in structure of ion channels. These are a major cause of shortening of APD and ERP. Therefore, the abnormal expression of ion channels is a material basis in the electrical remodeling of atrial myocardial cells after AF [20].

Our results showed the mRNA and protein expressions of L-type calcium channels α_1_c after 6 h rapid pacing had a decreasing trend, and the expressions of L-type calcium channels α_1_c reduced over time. The mRNA and protein expressions of potassium channel Kv4.3 decreased at 12 h after rapid pacing but thereafter remained stable and did not further reduce over time. In addition, the changes in the expression of L-type calcium channel α_1_c and potassium channel Kv4.3 were consistent with the findings in other models, but these changes were different from previously reported in the speed of changes. This may be attributed to following factors: ① The frequency of pacing is different in different experiments. In the present study, the pacing frequency was 600 beats/min. In the study of Yue et al. [6], the pacing frequency was 400 beats/min. The different pacing frequencies may present with differences in induction of ion channel remodeling, and rapid pacing may induce earlier remodeling. ② Different models were used in different experiments. In the studies of Grunnet et al. [8] and Yue et al. [6], animal models were used, while myocardial cells were used in the present study. ③ Animals of different species were used in different studies. In the goat AF model, the shortening of effective refractory period was earlier than that in dog AF model [21]. In addition, the occurrence of electrical remodeling in horse was later than that in goats and dogs.

Our results also showed that, after rapid pacing, the protein and mRNA expression of potassium channel Kv4.3 also decreased to different extents, which may not explain the shortening of effective refractory period and action potential cycle, because the decline in expression of potassium channel Kv4.3 and reduction of outward K^+^ flow will lead to extension of action potential cycle and effective refractory period. One possible hypothesis is that the reduced expression of potassium channel is attributed to the self-adaption of atrium. Brundel et al. [22] investigated the changes in the expression of potassium channel in AF patients, and results showed the mRNA and protein expression of potassium channel in persistent AF patients had a reducing trend, while only the protein expression of potassium channel reduced in the paroxysmal AF patients which suggests the presence of post-transcriptional regulation. This may be explained that rapid stimulation and increase in intracellular calcium may induce calcium overload as well as structural changes. Meanwhile, the expression of proteolytic enzymes can be increased in the atrial tissues and the neutral protease (such as calpains) be activated. These enzymes can lead to degradation of skeleton proteins, membrane proteins and regulatory protein, and also influence the expression of potassium ion channels.

In addition, to generate the cardiac action potential, in addition to inward sodium and calcium currents, 5 potassium currents are primarily involved: the inward-rectifier background current (IK1), the rapidly activating and inactivating transient outward current (Ito), and the ultrarapid (IKur), rapid (IKr), and slow (IKs) components of delayed rectifier currents [23]. More attention has been paid to IKr (KCNH2 gene expression) and Ito (KCND3 gene expression). KCNH2 encodes the α-subunit of the IKr channel, and membrane depolarization induced by strong inward currents produces a sequence of conformation changes within the channel that allows permeation of potassium ions [23]. The transient outward current (Ito) that mediates early (phase 1) repolarization and is conducted by the Kv4.3 pore-forming α-subunit encoded by KCND3 in humans remains central to the “the repolarization disorder” theory of the electrocardiographic and arrythmogenic manifestations of tachyarrhythmia [24]. Both of them result in shortening of action potential duration and atrial refractory period, facilitating multiple re-entrant circuits in AF.

## Conclusions

In summary, enzymatic digestion can be used to separate atrial myocardial cells, and differential adherent technique and incubation with BrdU be used to purify these atrial myocardial cells. The collected atrial myocardial cells meet the requirements for experiments. Pacing with electric field can be employed to establish in vitro rapid pacing model. The mRNA and protein expressions of the L-type calcium channel α_1_c and potassium channel Kv4.3 began to decrease to different extents after 6-h rapid pacing, suggesting that rapid pacing may induce the downward ion channel remodeling in atrial myocardial cells.

## Abbreviations

RT-PCR: Reverse transcription polymerase chain reaction; mRNA: Messenger RNA; AF: Atrial fibrillation; APD: Action potential duration; ERP: Endocardial return percentage; FBS: Fetal bovine serum.

## Competing interests

The authors declare that they have no competing interests.

## Authors’ contributions

YM and WD conceived of the study, and participated in its design and coordination and helped to draft the manuscript. QJ and JF participated in the design of the study and performed the biochemical analysis. XW carried out the biochemical analysis and drafted the manuscript. All authors read and approved the final manuscript.
