# Erratum: Expression changes of ionic channels in early phase of cultured rat atrial myocytes induced by rapid pacing

**DOI:** 10.1186/s13019-015-0210-4

**Published:** 2015-01-23

**Authors:** Wei Cheng, Qiang Ji, Hua Liu, Yunqing Mei, Xisheng Wang, Jing Feng, Wenjun Ding

**Affiliations:** 1 Department of Cardiovascular Surgery of Xinqiao Hospital of Third Military Medical University Chongqing, 83 Xinqiao Main Street, Chongqing, 400037 P.R. China; 2 Department of Thoracic Cardiovascular Surgery of Tongji Hospital of Tongji University Shanghai, 389 Xincun Rd, Shanghai, 200065 P.R. China; 3 Department of Cardiovascular Surgery of Zhongshan Hospital of Fudan University Shanghai, 180 Fenglin Rd, Shanghai, 200032 P.R. China

Following publication of this manuscript [1] it was discovered that Wei Cheng had been inadvertently missed from the author list. This has now been corrected in the author list above. The authors apologise for any inconvenience this may cause.
